# Detection of Retinoic Acid in Cosmetics Using Reactive Paper Spray Ionization Mass Spectrometry

**DOI:** 10.3390/molecules30091906

**Published:** 2025-04-25

**Authors:** Yuzhang Bao, Chenyu Wang, Na Zhang, Jie Li, Song Yuan, Liju Yu, Bin Di, Yang Liu

**Affiliations:** 1 National Institutes for Food and Drug Control, Beijing 102629, China; baoyzya@163.com (Y.B.); cpuwangchenyu@163.com (C.W.); zhangna@nifdc.org.cn (N.Z.); lijie@nifdc.org.cn (J.L.); yuansong@nifdc.org.cn (S.Y.); yuliju@nifdc.org.cn (L.Y.); 2 School of Pharmaceutical Sciences, China Pharmaceutical University, Nanjing 211100, China

**Keywords:** mass spectrometry, retinoic acid, cosmetics, chemical derivatization

## Abstract

Chromatography–mass spectrometry typically requires a time-consuming and costly pretreatment to detect illegal additives in cosmetics. Retinoic acid is classified as a prohibited additive in cosmetics by the European Union and China. Therefore, a rapid and convenient method is needed for its detection. In this study, a method for detecting retinoic acid using Reactive Paper Spray Ionization Mass Spectrometry was developed. *N*′-dimethylpiperazine was used as the derivatization reagent due to its ability to react with carboxyl functional groups at room temperature. Our results indicate that the derivatized retinoic acid compounds obtained using this method exhibited good linearity within the range of 0.0005~0.1 μg·mL^−1^, achieving a limit of detection of 0.107 ng·mL^−1^.

## 1. Introduction

Mass spectrometry (MS) is widely used across various fields because of its high sensitivity and precision [1,2,3]. In gas chromatography–mass spectrometry (GC-MS), the inherent physicochemical properties of retinoic acid—notably, its high polarity and low volatility—present significant analytical challenges. The technique primarily employs electron impact (EI) and chemical ionization (CI) sources. While EI ionization induces molecular fragmentation through high-energy electron bombardment, this process may degrade retinoic acid structures, compromising the quantitative accuracy. Although CI generates fewer fragments through reagent gas interactions, its analytical sensitivity remains constrained by ionization efficiency limitations. Liquid chromatography–mass spectrometry (LC-MS) methodologies typically utilize electrospray ionization (ESI) or atmospheric pressure chemical ionization (APCI) sources. Comparative analyses reveal that APCI demonstrates a superior matrix tolerance in complex cosmetic formulations, albeit with reduced sensitivity compared to ESI’s enhanced detection capabilities. Capillary electrophoresis–mass spectrometry (CE-MS) encounters practical limitations in cosmetic analysis due to its surfactant-induced electrophoretic instability and insufficient sensitivity for trace retinoic acid detection. Furthermore, all chromatographic–mass spectrometric approaches necessitate laborious sample preparation protocols, significantly increasing the analytical time requirements.

RPSI-MS (Reactive Paper Spray Ionization Mass Spectrometry) uses an ESI ion source. Since retinoic acid contains only C, H, and O elements and is not easily ionized, derivatization is performed to enhance its mass spectrometric response. The ESI ion source is sensitive to matrix effects, and substances like oils and emulsifiers in cosmetics can suppress ionization. However, the paper substrate can selectively adsorb interfering substances such as oils, reducing the ion suppression. Moreover, RPSI-MS can simplify the pretreatment and save time, making it suitable for the rapid screening of the illegal additive retinoic acid in cosmetics.

Consequently, derivatization has become an essential method for enhancing the sensitivity of MS analysis. Chemical derivatization involves introducing active groups into a compound, which alters its physical and chemical characteristics, thereby enhancing its detectability. Chemical derivatization is extensively used in drug analysis [4], biometabolomics [5,6,7,8], environmental monitoring [9,10,11] and food safety detection [12,13,14].

Paper spray ionization mass spectrometry (PSI-MS) [15] was introduced in 2010 as a type of ambient ionization mass spectrometry (AMS) and has been widely employed in recent years. Despite its broad applications, PSI-MS has several limitations. For example, PSI-MS detects compounds under normal pressure conditions, making it less sensitive than traditional MS techniques. This shortcoming can be mitigated by chemical derivatization, which enhances the ionization capabilities of poorly responsive compounds, thereby improving their detection using PSI-MS. Consequently, the sensitivity of these derivatized compounds can be improved to levels similar to those achieved with traditional MS detection. Moreover, compared with traditional MS, PSI-MS does not require complex sample pretreatment, uses fewer samples, and saves time and costs.

Reactive Paper Spray Ionization Mass Spectrometry (RPSI-MS) represents an innovative advancement in PSI-MS methodology. In contrast to conventional PSI-MS, which requires the offline preparation of derivatized solutions in centrifugal tubes followed by depositing them onto triangular cellulose substrates for the MS analysis, RPSI-MS integrates chemical derivatization directly into the ionization process. In this approach, the analyte is mixed with a catalytic reagent, and the resulting solution is applied to a pre-treated triangular paper substrate impregnated with derivatization agents. During ionization, solvent evaporation progressively enriches the derivatives, thereby amplifying the detection sensitivity beyond the capabilities of conventional PSI-MS. Currently, RPSI-MS is widely employed to enhance the detection sensitivity via chemical derivatization, with applications in aldehyde analysis [16], metabolomics [17,18], estrogen detection [19], and chemical reaction monitoring [20,21,22].

People are paying increasing attention to skincare. Consequently, the demand for skin whitening, acne treatment, anti-aging products, and other skin treatments is increasing, driving the growth of the efficacious skincare products industry. Various ingredients have been developed and incorporated into skincare products. For example, nicotinamide [23] and vitamin C [24] are used for whitening, Centella asiatica extracts [25] and ceramides [26] are employed for soothing and repair, and astaxanthin [27] and retinol [28] are used in anti-aging products.

**Figure 1:**
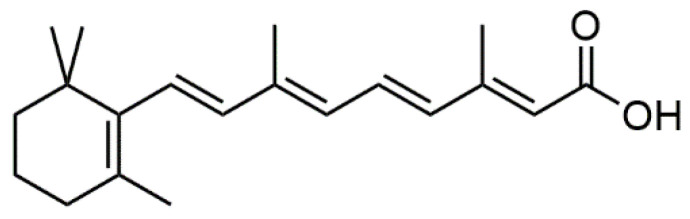
The structure of retinoic acid.

Several unscrupulous merchants have taken advantage of the rapid growth of the skincare products market to pursue profits. Retinoic acid (Figure 1) inhibits the activity of catalytic enzymes, such as tyrosine hydroxylase, dopa oxidase, and dihydroxyindole, leading to a reduction in melanin formation and skin pigmentation. When the skin is damaged by external factors, such as UV radiation, retinoic acid can help protect and repair the skin. Currently, it is primarily used to treat acne [29,30,31], particularly blackheads, sunlight- or pharmaceutical-induced skin atrophy, ichthyosis, hyperpigmentation, and psoriasis [32,33]. However, pharmacological research has shown that retinoic acid has strong teratogenicity and can irritate human skin. Therefore, it can only be used as a prescription drug. It is included among the banned additives in cosmetics in the Catalogue of Prohibited Cosmetic Products of the Chinese Safety and Technical Standards (2015) [34] and in Regulation (EC) No. 1223/2009 of the European Parliament and Council [35].

Given the risks associated with the illegal addition of retinoic acid to skincare products, the accurate determination of its content is crucial. The commonly used methods for detecting retinoic acid include liquid chromatography [36] (LC) and MS [37]. However, these methods are time-consuming and expensive; therefore, a faster detection method is required. In this study, we developed and evaluated an RPSI-MS method for detecting retinoic acid in skincare products. This method combines the advantages of PSI-MS with online derivatization, enabling simple, rapid, and highly sensitive detection.

## 2. Results and Discussion

### 2.1. Online Derivatization Reaction

Online derivatization involves adding a derivatization reagent and a sample separately to the same carrier for reaction. In contrast to bulk reactions, where a specific volume of the derivatization reagent is mixed with the sample in advance for derivatization, online derivatization occurs in a small-volume “reactor”. Research has shown that small-volume reactors have a larger surface-area-to-volume ratio and a higher solution concentration as the solvent evaporates, making the online derivatization faster and more efficient than bulk reactions [38,39,40,41,42,43].

RPSI-MS involves adding derivatization reagents and compounds onto a triangular paper substrate. Driven by the flow of a spray solvent under a high electric field, a thin layer of liquid film forms on the paper substrate. This spray solvent then extracts the derivatized compounds from the paper substrate, generating microdroplets that form an electrojet, which are subsequently introduced into the MS for detection. These tiny droplets contain unreacted samples, derivatization reagents, and catalytic reagents. The concentrations of these components are higher in smaller droplets, enabling the unreacted compounds to react at ultrahigh rates. These reacted compounds enter the MS alongside the derivatized compounds that have already reacted on the paper, resulting in an improved MS response. Notably, the rates of the microvolume reactions on the paper substrate and the microdroplet reactions are higher than those of the bulk-phase reactions [44,45].

**Figure 2:**
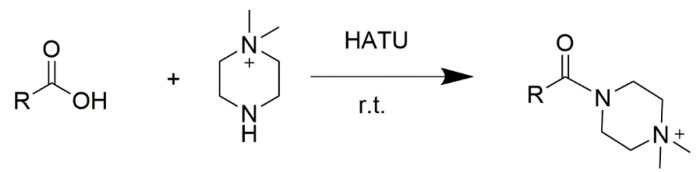
The reaction between a carboxyl compound and DMPI.

In this study, we performed the online derivatization of retinoic acid and the internal standard fenbufen and detected their derivatized compounds (Figures S1–S7) using PSI-MS. *N*, *N*′-dimethylpiperazine (DMPI) [46] was selected as the derivatization reagent because it can derivatize the carboxyl groups of compounds at room temperature with a rapid reaction rate (Figure 2). Prior to derivatization, the carboxyl group on retinoic acid was activated by alkali and then condensed with onium salt condensers, such as 2-(7-Azabenzotriazol-1-yl)-1,1,3,3-tetramethyluronium hexafluorophosphate (HATU) and O-Benzotriazole-*N*,*N*,*N*′,*N*′-tetramethyluronium hexafluorophosphate (HBTU). Studies have demonstrated that HATU exhibits better reactivity and a higher yield than HBTU; therefore, we chose HATU as the catalyst for the acylamide condensation [47].

### 2.2. Optimization of Derivatization Conditions

Cosmetics contain various ingredients, including surfactants, efficacious ingredients, greases, preservatives, and fragrances. These substances may also react with the derivatization reagent; therefore, the reaction system must contain an excess of DMPI. Overloading the triangular paper can cause a blockage at the tip of the paper, which adversely affects the spraying effect; therefore, we optimized the concentration and volume of DMPI on the paper substrate. The results indicate that the optimal MS response was achieved when the concentration of DMPI was 3 mg·mL^−1^ and the volume of DMPI was 3 μL.

**Figure 3:**
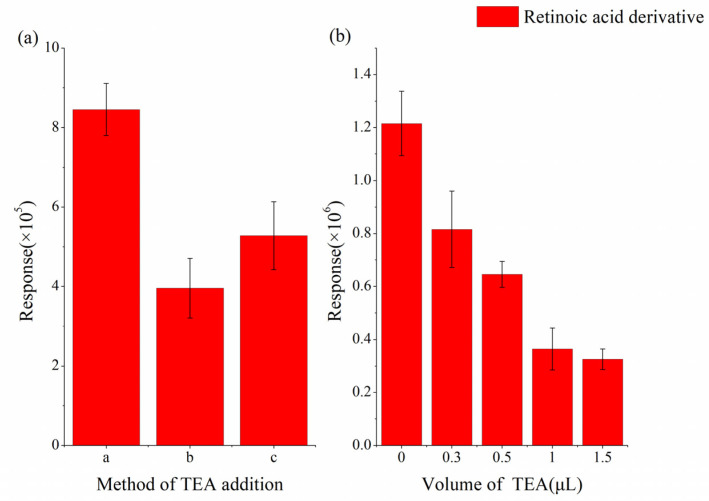
MS response versus (**a**) different methods of adding TEA (from left to right): without additional TEA, mixture of derivatization reagent and TEA solution, and mixture of retinoic acid and TEA solution. (**b**) MS response versus volume of TEA added directly to paper.

The derivatization of carboxyl groups requires the addition of alkali; therefore, we examined the effect of adding TEA to the mixture of retinoic acid and DMPI. Considering that a TEA solution had already been added during the dissolution of the retinoic acid and DMPI to enhance their solubility, the results in Figure 3 show that the system already contained sufficient alkali, and additional TEA reduced the MS response due to the ion suppression effect.

### 2.3. Optimization of the Drying Time for Paper Spray MS

**Figure 4:**
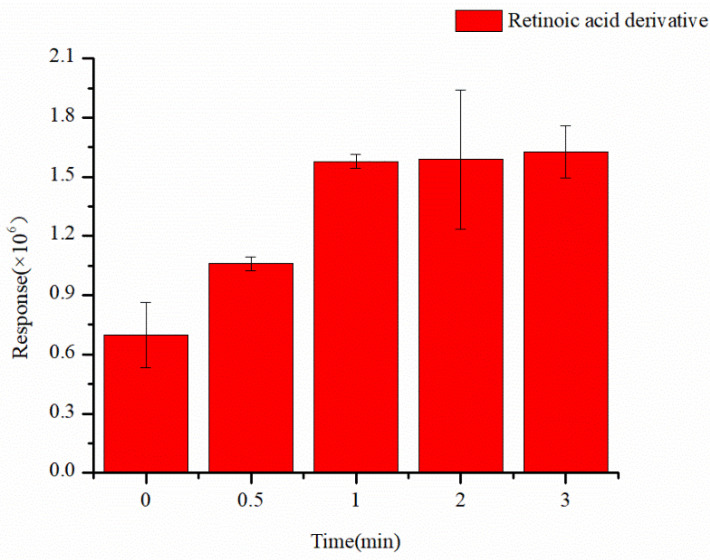
Effect of paper placement time on reaction process of RPSI-MS.

The derivatization of retinoic acid requires a specific reaction time on the paper substrate. We optimized the drying time of the triangular paper, which contained DMPI and a mixture of retinoic acid and HATU. The results (Figure 4) indicate that a drying time of 1 min yielded the optimal MS response, with no significant change observed after 1 min. Thus, we selected 1 min as the optimal drying time for the paper substrate.

### 2.4. Sensitivity and Linearity

**Figure 5:**
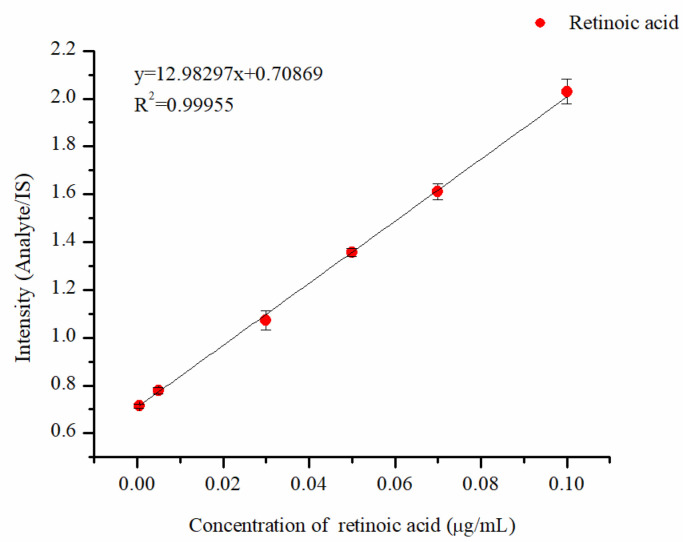
Linearity of retinoic acid derivative.

The prepared mixed solution, ranging from low to high concentrations, was added to a triangular paper substrate containing DMPI for the online derivatization and subsequent MS detection. Each concentration was tested with three repeated injections. The ratio of the peak area of the analyte to that of the internal standard was used as the vertical coordinate, while the concentration of retinoic acid served as the horizontal coordinate to plot the linear curve. The results show that the retinoic acid-derived compounds exhibited excellent linearity within the range of 0.0005~0.1 μg·mL^−1^, with a fitting equation of y = 12.98x + 0.7087 and a correlation coefficient (R^2^) of 0.9996 (Figure 5). The limit of detection (LOD), calculated using D = 3δ/S, where D represents LOD, δ represents the standard deviation of six injections of the blank solution, and S represents the slope of the linearity, was 0.107 ng·mL^−1^, and the limit of quantification (LOQ) was 0.356 ng·mL^−1^ (LOQ = 10δ/S).

### 2.5. Precision of the Experiment

Three solutions with different concentrations (0.001, 0.01, and 0.1 μg·mL^−1^) within the linear range were subjected to online derivatization, with five parallel measurements conducted for each concentration. The average concentrations were calculated to be 0.001011, 0.01012, and 0.1007 μg·mL^−1^, respectively, and their RSDs were all less than 10%.

### 2.6. Sample Recovery Experiment

After adding 2 mg of a blank sample to 200 μL of different concentrations of the retinoic acid solution (0.001, 0.01, and 0.1 μg·mL^−1^), the mixture was sonicated for 1 min. In addition, 200 μL of an internal standard solution and 80 μL of HATU (1 mg·mL^−1^) were added. Subsequently, the mixture was vortexed for 30 s and applied onto the triangular paper containing DMPI for the online derivatization. The resulting sample was then introduced into the MS for detection.

Retinoic acid recovery solutions with low (0.001 μg·mL^−1^), medium (0.01 μg·mL^−1^), and high (0.1 μg·mL^−1^) concentrations were subjected to online derivatization for the MS, and three parallel determinations were made for each concentration. The average recoveries were calculated as 96.29%, 100.09%, and 98.78%, respectively, and the RSDs were all below 10% (Table 1).

### 2.7. Complex Matrix Sample Detection

The applicability of the online derivatization method was assessed by adding quantitative amounts of the target compounds to complex matrices (creams containing glycerol, triglycerides caprylate, cetearyl stearyl alcohol, polydimethylsiloxane, and other matrices) and performing MS after the online derivatization. The results indicate that this method can be used to semi-quantitatively detect retinoic acid added to complex matrices.

## 3. Materials and Methods

### 3.1. Materials and Reagents

All of the reagents were used as obtained without undergoing additional purification. The methanol was obtained from Merck (Darmstadt, Germany). The acetonitrile was purchased from Fisher Scientific (Waltham, MA, USA). The triethylamine was sourced from Taitan Science & Technology (Shanghai, China). The retinoic acid and fenbufen were purchased from the National Institute for Food and Drug Control (Beijing, China). The HATU was purchased from TCI Chemicals (Shanghai, China). The grade 1 chromatographic paper was obtained from Whatman (St. Venage, UK). The DMPI was synthesized in the laboratory following a previously published procedure [46]. The structure of the DMPI matched that described in the literature: ^1^H NMR (D_2_O, TMS): δ 3.41 (6H, s, CH_3_), δ 3.79–3.87 (8H, m, CH_2_); HRMS: C_6_H_15_N_2_^+^ (calculated: 115.1229, found: 115.1230).

### 3.2. Instruments

All of the High Performance Liquid Chromatography (HPLC) experiments were performed using an Agilent 1290 system coupled with a 6495 triple quadrupole MS (Palo Alto, CA, USA). An analytical balance with a precision of one part per million (METTLER TOLEDO, Zurich, Switzerland), a KQ-500DA CNC ultrasonic cleaner (Kunshan Ultrasonic Instrument Co., Ltd., Kunshan, China), and an HB-Z303-1AC high-voltage DC power supply (Tianjin Hengbo High Voltage Power Supply Factory, Tianjin, China) were used.

### 3.3. Sample Preparation

The standard stock solution (100 μg·mL^−1^) was prepared using a two-step dilution protocol. Initially, 10 mg of retinoic acid was dissolved in 1 mL of a triethylamine–acetonitrile solution (1 mol·L^−1^) in a 10 mL volumetric flask, and the volume was then adjusted with pure acetonitrile. For the secondary dilution, a 1 mL aliquot from the primary solution was transferred to a fresh 10 mL volumetric flask and reconstituted with acetonitrile to achieve the target concentration.

The linear standard solutions were prepared by diluting the stock solution with acetonitrile to obtain concentrations of 0.0005, 0.005, 0.03, 0.05, 0.07, and 0.1 μg·mL^−1^.

The internal standard working solution (0.1 μg·mL^−1^) was prepared through the sequential dilution of fenbufen. Initially, 10 mg of the standard was transferred into a 10 mL volumetric flask and dissolved in 1 mL of a triethylamine–acetonitrile mixture (1 mol·L^−1^). The volume was then adjusted with acetonitrile to yield a 1 mg·mL^−1^ stock solution. Subsequently, this primary solution was diluted 10,000-fold with acetonitrile to attain the target concentration.

To prepare a triangular paper substrate with DMPI, the cut paper was placed on a slide, and 3 μL of DMPI solution (3 mg·mL^−1^) was applied to it. The paper was allowed to dry naturally before use.

### 3.4. Derivative Reaction

An HATU solution (1 mg·mL^−1^, 20 μL) was added to 50 μL of the linear standard solution and 50 μL of the internal standard solution. The resulting mixture was vortexed for 30 s and then applied onto the triangular paper containing DMPI for the online derivatization and RPSI-MS analysis.

### 3.5. MS Conditions

**Figure 6:**
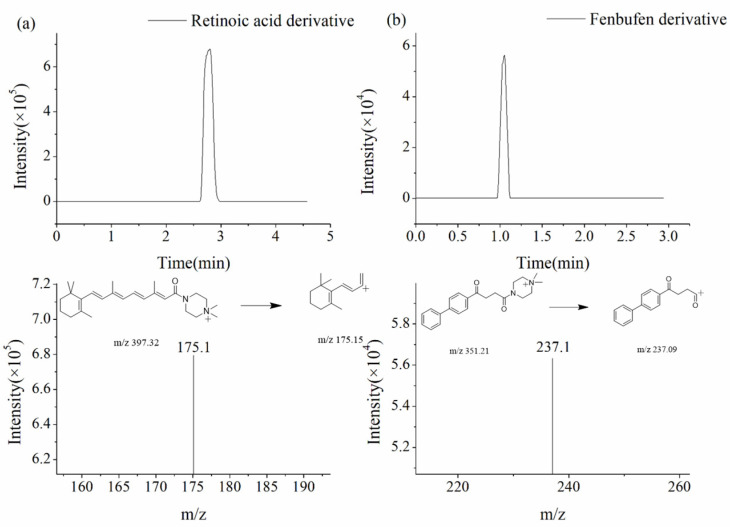
Fragmentation pathways of mass spectra for (**a**) retinoic acid derivative (MRM mode) and (**b**) fenbufen derivative (MRM mode).

An Agilent 1290 HPLC system coupled with a 6495 triple quadrupole MS was used, with an Agilent Jet Stream Electrospray Ionization (AJS ESI) ion source in positive ion scan mode. The gas and sheath gas temperatures were set to 100 °C, and their flow rates were maintained at 11 L·min^−1^. The detection was performed in the multiple reaction monitor (MRM) mode. The cell accelerator voltage was set to 6 V, and the collision energy (CE) was set to 24 eV. The quantitative ion pairs were 397.3→175.1 for retinoic acid and 351→237.1 for fenbufen (Figure 6).

### 3.6. Derivatization of Retinoic Acid Using Paper Spray MS

**Figure 7:**
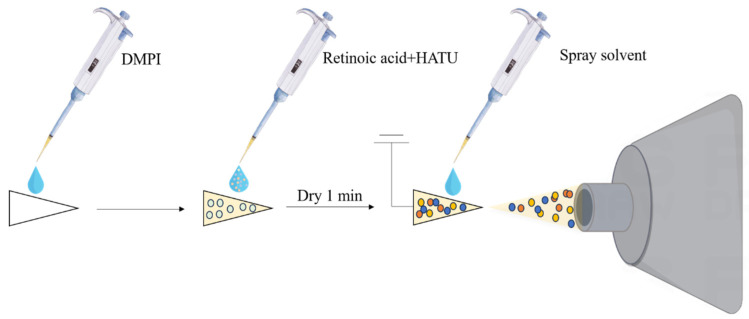
Workflow of online derivatization process for retinoic acid.

The dried triangular paper containing DMPI was fixed with a copper clip, with the tip of the paper facing the cone hole of the MS ion source. Subsequently, 2.5 μL of the linear sample solution was added to the triangular paper for the online derivatization, and the paper was dried for 1 min. After adding 20 μL of the spray solvent under a 1.0 kV external electric field, the spray solvent carried the derivative to the tip of the paper and formed an electrojet that entered the MS for detection (Figure 7).

## 4. Conclusions

In this study, we employed RPSI-MS to detect retinoic acid derivatives, achieving an LOQ of 0.356 ng·mL^−1^. Non-derivatized retinoic acid was detected via PSI-MS with an LOQ of 0.237 μg·mL^−1^ (Figure S8). The sensitivity of the retinoic acid detection using RPSI-MS was more than 660 times greater than that of the non-derivatized retinoic acid detection using PSI-MS. Furthermore, RPSI-MS exhibited an approximately 12-fold higher sensitivity compared with PSI-MS for detecting retinoic acid derivatives [48]. The sensitivity of RPSI-MS for detecting retinoic acid in cosmetics was approximately 941 times higher than that of HPLC, as reported in the literature [36]. Moreover, these results are comparable to those achieved with Ultra-High Performance Liquid Chromatography—Time of Flight—Mass Spectrometry (UHPLC–ToF–MS) [37]. RPSI-MS effectively detected retinoic acid in cosmetics, offering an approach that combines the advantages of PSI-MS and online derivatization. PSI-MS does not require sample pretreatment, and the samples can be directly introduced into the MS for detection after sufficient mixing with the derivatization reagent, saving both time and costs. Moreover, the online derivatization reaction exhibits a stronger response, which can be attributed to the high surface-area-to-volume ratio of the small reaction system, promoting the reactions between the derivatization reagent and the analyte. Therefore, as a novel technology, RPSI-MS shows promise for applications in detecting compounds with low MS responses.

Despite certain advancements achieved, this study presents several limitations requiring further investigation. Firstly, the experimental validation was exclusively conducted on facial creams using Reactive Paper Spray Ionization Mass Spectrometry (RPSI-MS), while essential verification tests—particularly spiked recovery experiments—remain to be performed for other cosmetic matrices, such as facial masks and essence liquids. This omission necessitates additional validation to fully evaluate the broader applicability of the proposed methodology. Secondly, given that retinoic acid is typically present at trace levels in illegally adulterated cosmetics, the current analytical challenges primarily stem from matrix interference effects. Future investigations should prioritize the development of effective matrix cleanup protocols to enhance the instrumental sensitivity for ultratrace detection. Thirdly, establishing streamlined sample pretreatment procedures specifically optimized for retinoic acid extraction and purification would significantly improve the mass spectrometric response characteristics, thereby augmenting the overall method sensitivity.

Notwithstanding these limitations, this work establishes a novel framework for the rapid screening of retinoic acid in cosmetic products. Our ongoing research focuses on engineering storable triangular paper substrates pre-loaded with derivatization reagents. This technological advancement aims to facilitate the seamless integration of PSI with portable mass spectrometry platforms, ultimately enabling field-deployable analytical solutions for regulatory inspections.

## Figures and Tables

**Table 1:** Detection of retinoic acid in complex matrices.

Chemical Compound	Added (μg·mL^−1^)	Found (μg·mL^−1^)	Recovery Rate (%)	Average Recovery Rate (%)	RSD (%) (N = 3)
Retinoic acid	0.001	0.0009789	97.89	96.29	1.77
		0.0009449	94.49		
		0.0009649	96.49		
	0.01	0.01039	103.9	100.9	4.25
		0.009623	96.23		
		0.01025	102.5		
	0.1	0.09847	98.47	98.78	1.51
		0.09746	97.46		
		0.1004	100.4		

## Data Availability

The data presented in this study are available on request from the corresponding author.

## Supplementary Materials

The following supporting information can be downloaded at https://www.mdpi.com/article/10.3390/molecules30091906/s1: Figure S1: Mass spectrum of retinoic acid (product ion scan mode); Figure S2: Mass spectra of retinoic acid derivatives (product ion scan mode, online derivatization); Figure S3: Mass spectrum of retinoic acid (MRM mode); Figure S4: Mass spectra of retinoic acid derivatives (MRM mode); Figure S5: Mass spectra of retinoic acid derivatives (MRM mode, online derivatization); Figure S6: Mass spectrum of fenbufen (product ion scan mode); Figure S7: Mass spectra of fenbufen derivatives (product ion scan mode; online derivatization); Figure S8: Linearity of retinoic acid without derivatization.

## Abbreviations

The following abbreviations are used in this manuscript:

LC	Liquid chromatography
MS	Mass spectrometry
TEA	Triethylamine
DMPI	*N*,*N*′-dimethylpiperazine
HATU	2-(7-Azabenzotriazol-1-yl)-1,1,3,3-tetramethyluronium hexafluorophosphate
HBTU	O-Benzotriazole-*N*,*N*,*N*′,*N*′-tetramethyluronium hexafluorophosphate
PSI-MS	Paper spray ionization mass spectrometry
AMS	ambient ionization mass spectrometry
RPSI-MS	Reactive Paper Spray Ionization Mass Spectrometry
LOD	limit of detection
LOQ	limit of quantification
HPLC	High Performance Liquid Chromatography
AJS ESI	Agilent Jet Stream Electrospray Ionization
CE	collision energy
UHPLC–TOF–MS	Ultra-High Performance Liquid Chromatography—Time of Flight—Mass Spectrometry
