# Supplementary material for: Detection of Retinoic Acid in Cosmetics Using Reactive Paper Spray Ionization Mass Spectrometry
Source: Molecules. 2025 Apr 25;30(9):1906. doi: 10.3390/molecules30091906 (PMC12073399; doi:10.3390/molecules30091906)
Supplement: Supplementary file 1 [file molecules-30-01906-s001.zip › molecules-3541880-supplementary.pdf]

Supporting Information

Detection of Retinoic acid in Cosmetics Using Reactive Paper Spray Ionization Mass Spectrometry

Yuzhang Bao[1,2], Chenyu Wang[1,2], Na Zhang[1], Jie Li[1], Song Yuan[1], Liju Yu[1], Bin Di[2*] and Yang Liu [1*]

1 National Institutes for Food and Drug Control, Beijing 102629, China;
2 School of Pharmaceutical Sciences, China Pharmaceutical University, Nanjing, 211100; China;
* Correspondence: dibin@cpu.edu.cn (B.D.); yangliu@nifdc.org.cn (Y.L.);

Table of Content

Figure S1.Mass spectrum of retinoic acid(Product ion scan mode)…………….3

Figure S2.Mass spectrum of retinoic acid derivative(Product ion scan mode；online derivatization)…………………………..…………………………….3

Figure S3. Mass spectrum of retinoic acid (MRM mode)………...………...…4

Figure S4. Mass spectrum of retinoic acid derivative (MRM mode)….….……4

Figure S5. Mass spectrum of retinoic acid derivative (MRM mode; online derivatization)…………………………………………………………………5

Figure S6.Mass spectrum of Fenbufen(Product ion scan mode)……………….5

Figure S7.Mass spectrum of Fenbufen derivative (Product ion scan mode; online derivatization)……………………………………………………………6

Figure S8. The linearity of the retinoic acid without derivatization……………6

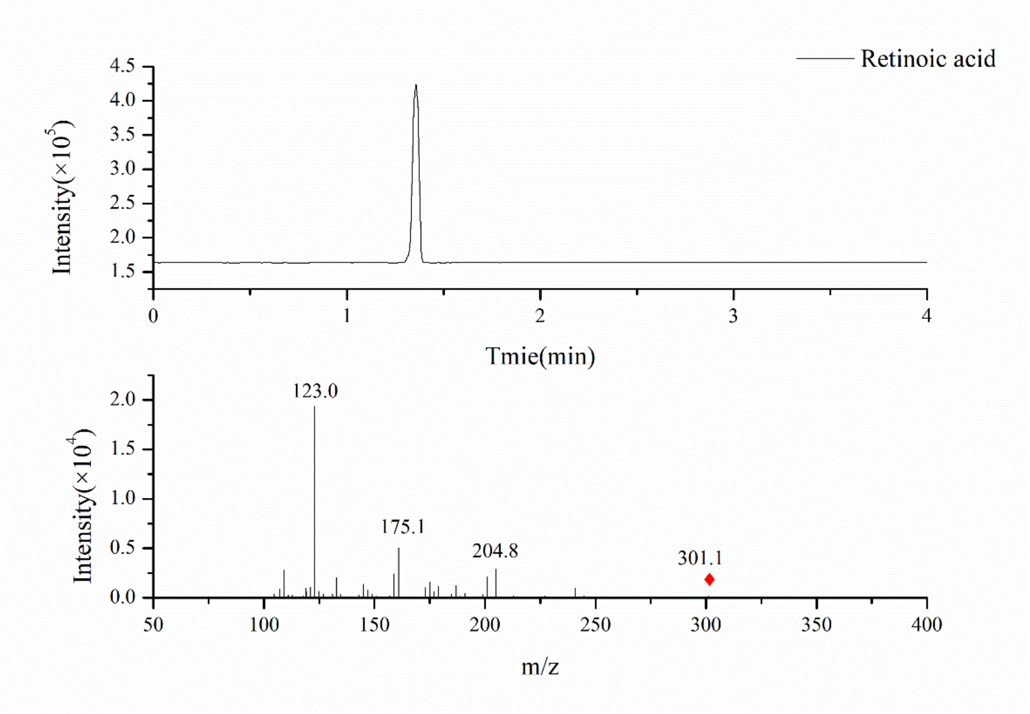

Figure S1. Mass spectrum of retinoic acid without derivatization (Product ion scan mode)

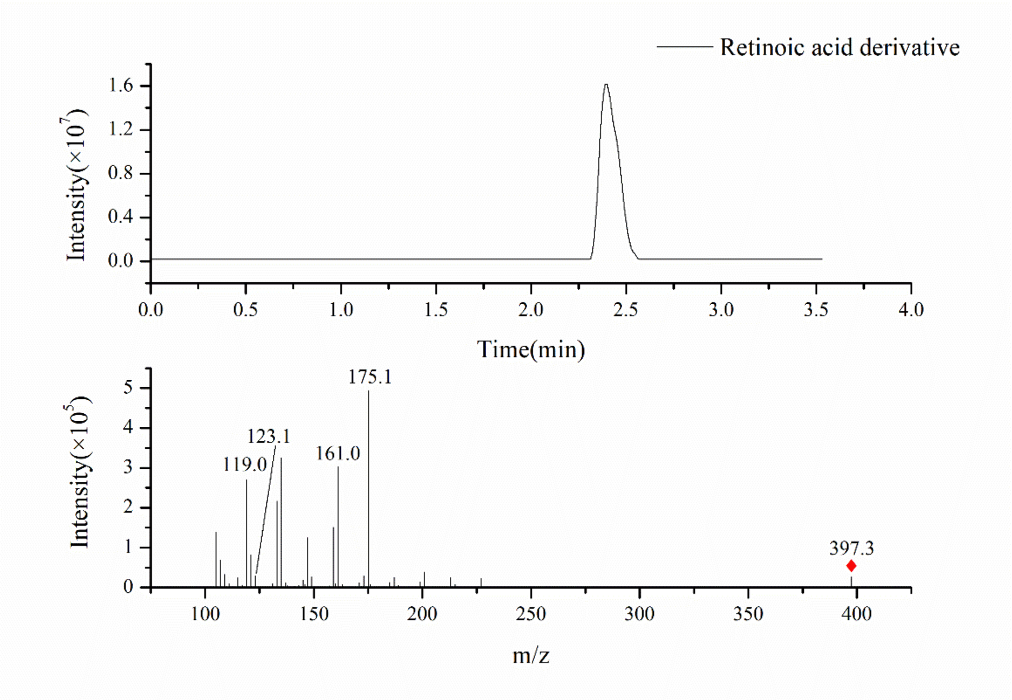

Figure S2.Mass spectrum of retinoic acid derivative (Product ion scan mode; online derivatization)

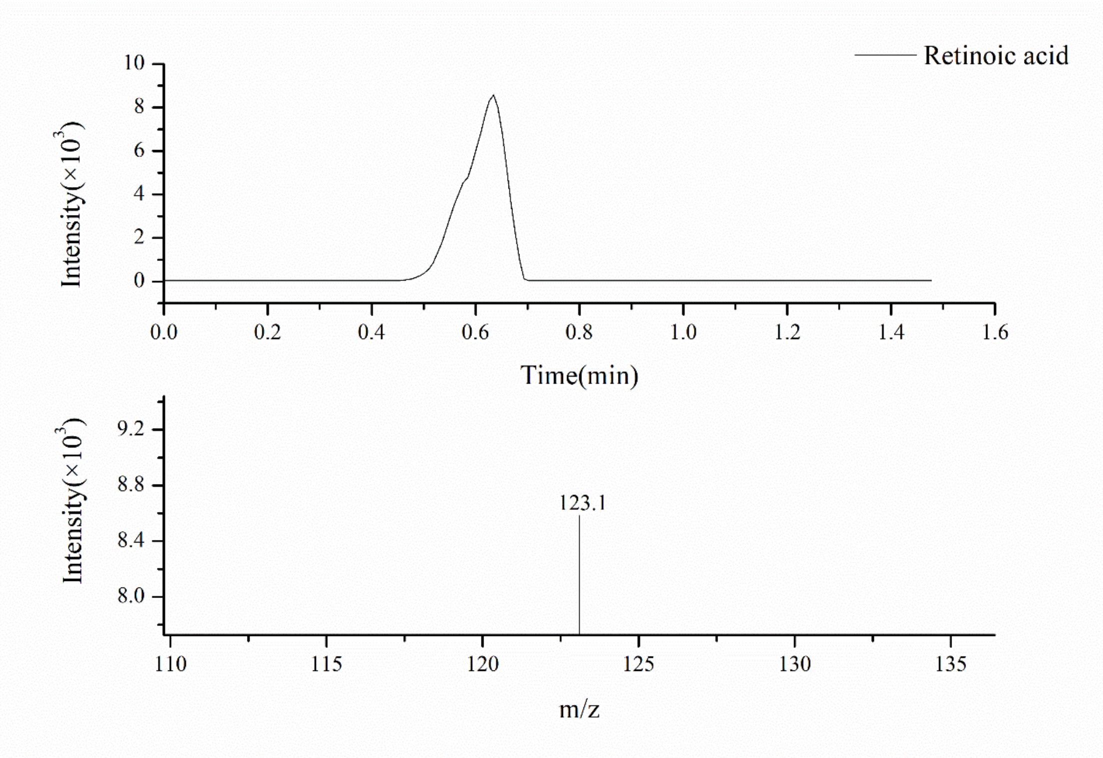

Figure S3. Mass spectrum of retinoic acid(MRM mode)

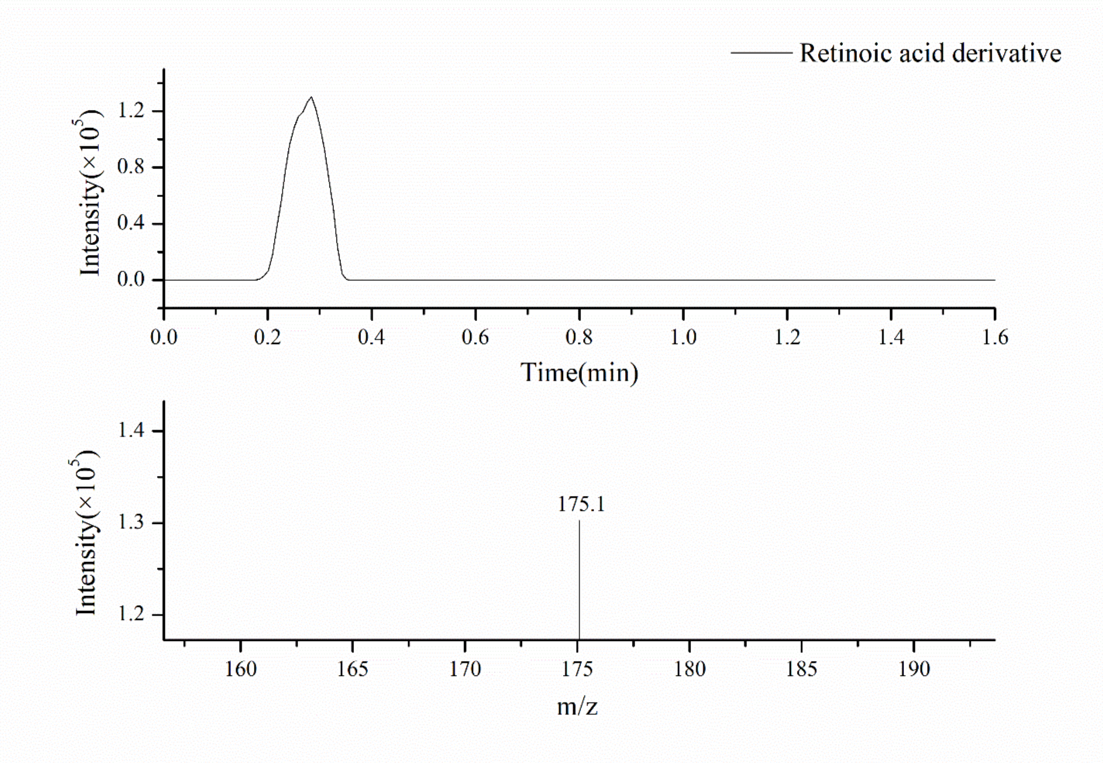

Figure S4. Mass spectrum of retinoic acid derivative (MRM mode)

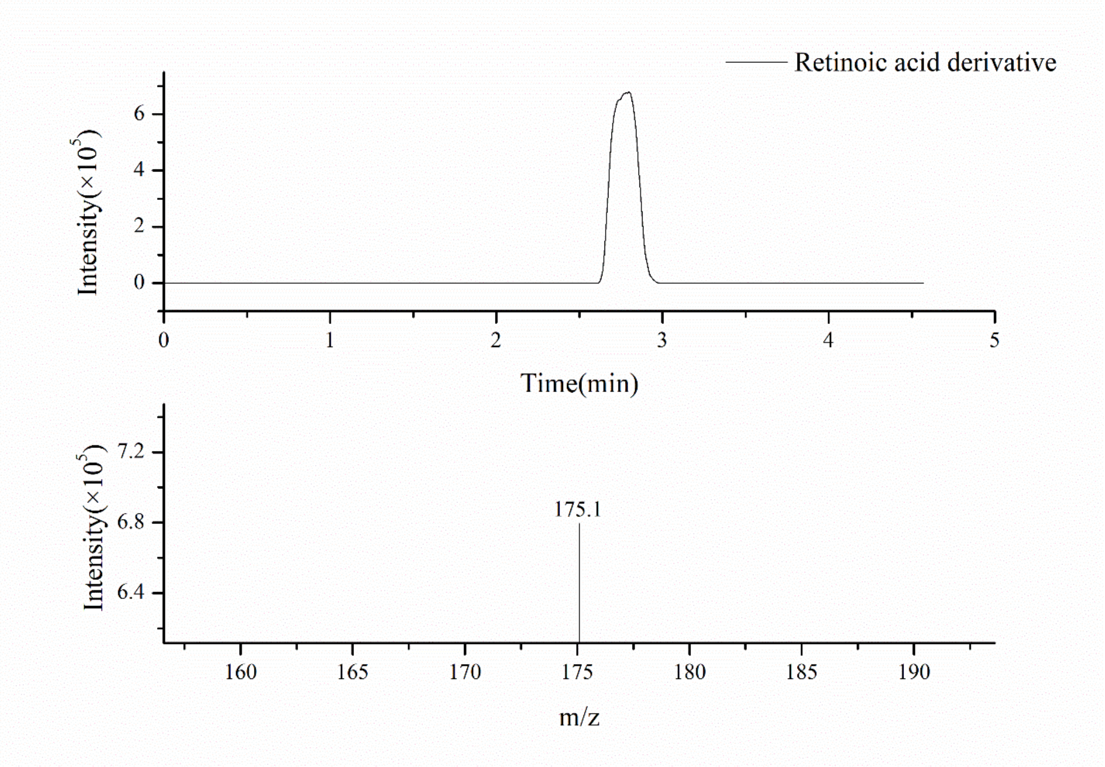

Figure S5. Mass spectrum of retinoic acid derivative (MRM mode; online derivatization)

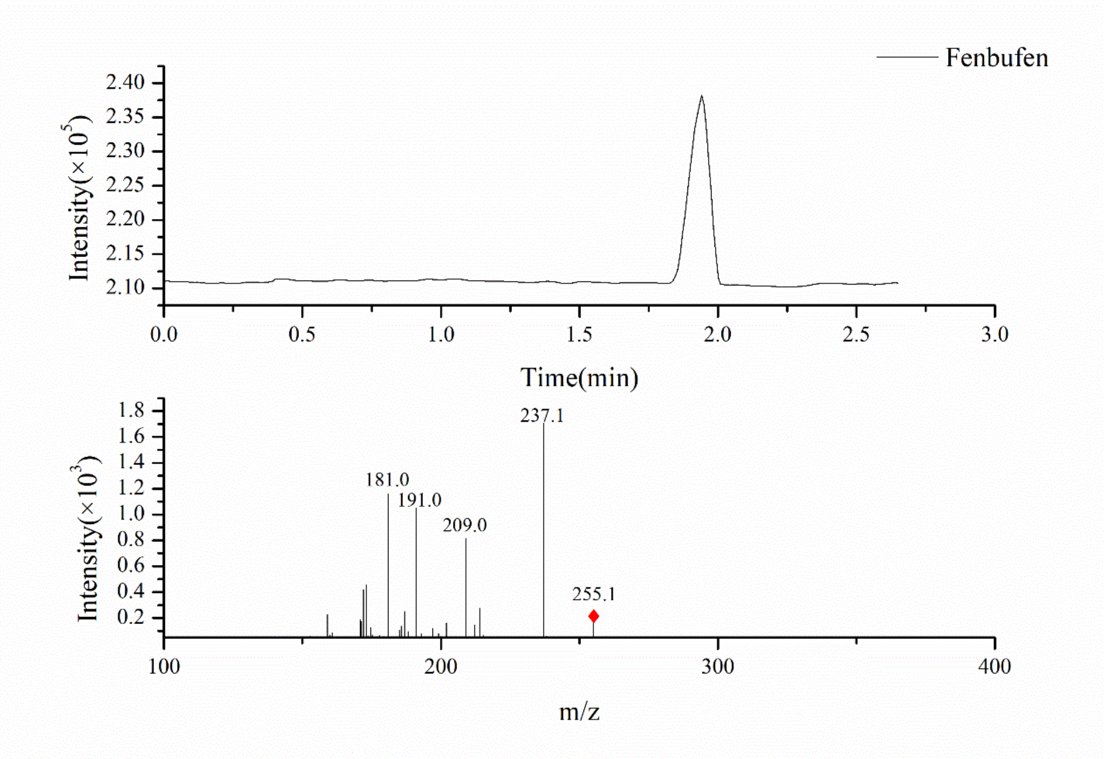

Figure S6. Mass spectrum of Fenbufen(Product ion scan mode)

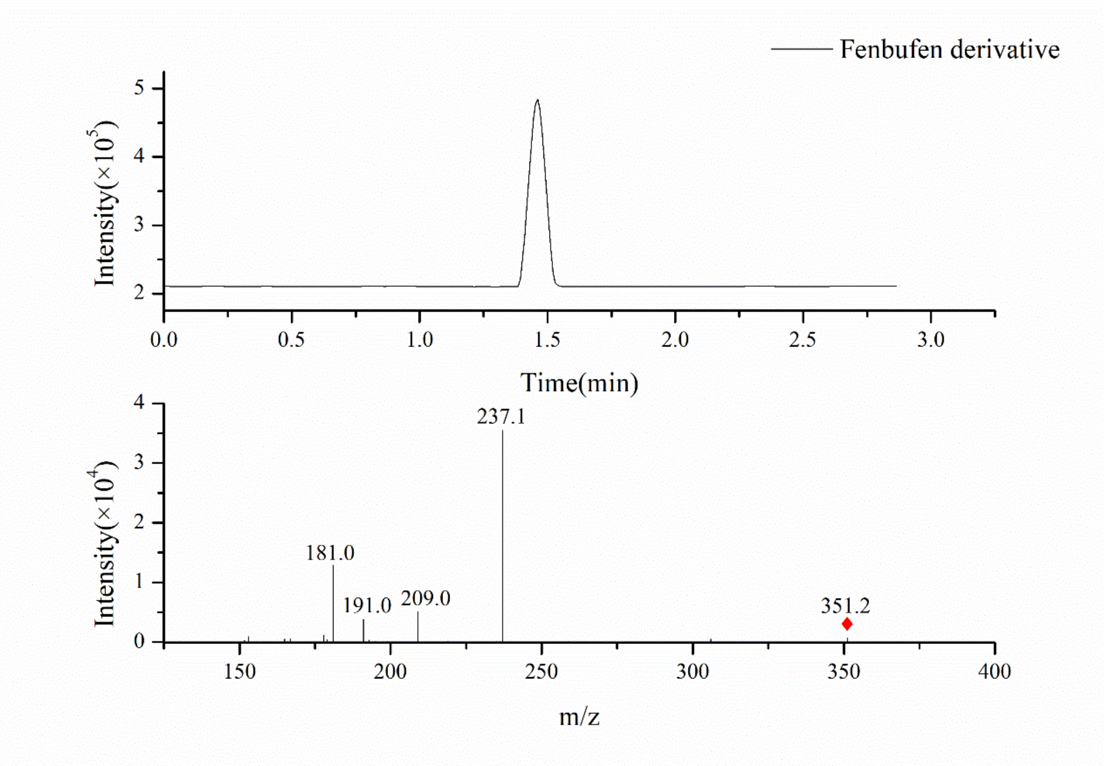

Figure S7.Mass spectrum of Fenbufen derivative (Product ion scan mode; online derivatization)

A concentration gradient of underivatized retinoic acid standard solutions (0.1-20 μg·mL^{-1}) was analyzed in triplicate to establish method linearity. Calibration curves were generated by plotting the analyte-to-internal standard response ratio (Y-axis) against corresponding retinoic acid concentrations (X-axis), demonstrating satisfactory linearity ($y = 0.00844x + 0.02147$, $R^2 = 0.99376$) as illustrated in Figure S8. Sensitivity thresholds were determined through statistical calculations, with the limit of detection (LOD = $3\sigma/S$) and quantification (LOQ = $10\sigma/S$) reaching 0.071 μg·mL^{-1} and 0.237 μg·mL^{-1} respectively, where σ represents the standard deviation of six blank injections and S denotes the calibration curve slope.

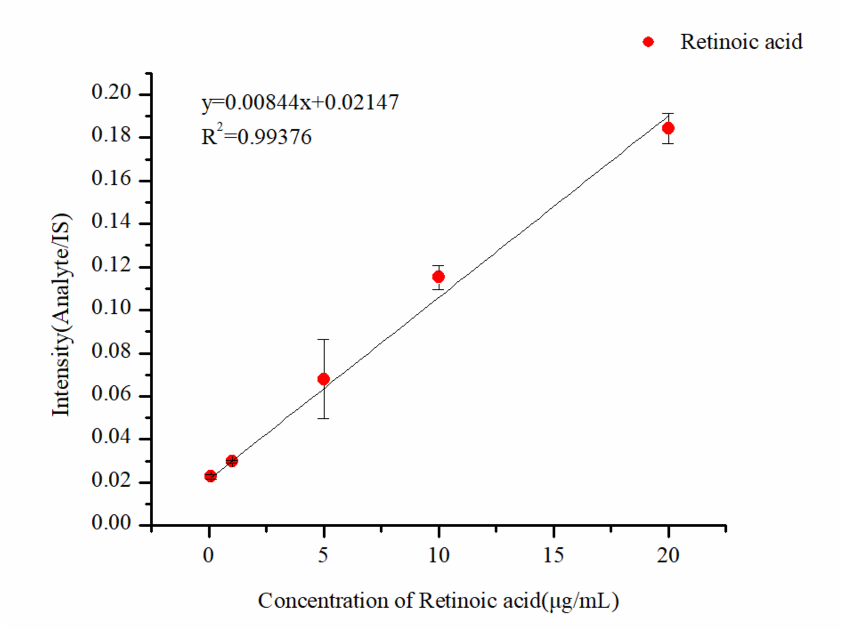

Figure S8.The linearity of the retinoic acid without derivatization
